# Pre-Ramadan Consultation: Does a Physician's Religious Belief and Specialty Matter?

**DOI:** 10.7759/cureus.33209

**Published:** 2023-01-01

**Authors:** Rabih M Abou Leila, Tamer Kolaib, Tarek Chreih

**Affiliations:** 1 Family Medicine, American Mission Hospital, Manama, BHR; 2 Family Medicine, Primary Health Care Corporation, Doha, QAT; 3 Family Medicine, Prince Sultan Military Medical City, Riyadh, SAU

**Keywords:** ramadan diet plan for weight loss, ckd patients and ramadan, ramadan fasting and dm, guidance and counselling, fasting ramadan

## Abstract

Background

A pre-Ramadan consultation is a practical approach to optimize the care of patients with chronic conditions before the month-long fast. The present study aims to assess healthcare professionals' knowledge, attitude, and practice (KAP) toward pre-Ramadan counseling in Arab countries and assess the effects of physicians' specialty and religious beliefs on their KAP.

Method

An online cross-sectional survey was conducted to assess physicians’ KAP toward pre-Ramadan consultation and management of patients with pre-existing health issues before Ramadan. Each participant got three scores: (1) knowledge score, (2) attitude score, and (3) practice score. A one-way ANOVA and post hoc tests were performed to detect the differences in physicians' KAP toward pre-Ramadan consultation with their specialties and religious backgrounds.

Result

Most of the participants did not use pre-Ramadan consultation timely (Only two of the 200 subjects did). The mean values of the physicians' scores were as follows: the knowledge score was 7.8 out of 17, the attitude score was 2.28 out of 4, and the practice score was 4.33 out of 11. However, post hoc tests showed that family physicians were more knowledgeable regarding pre-Ramadan consultation than other specialties. Moreover, Muslim participants achieved better attitude and practice scores than non-Muslim participants.

Conclusion

Most of the participants did not offer pre-Ramadan consultation timely. The attitudes and practices toward pre-Ramadan consultation were statistically different between Muslim and non-Muslim doctors. The findings of this study suggest that improving physicians' pre-Ramadan consultation knowledge is imperative to optimize the care of patients before Ramadan. Moreover, improving the attitude and practice of non-Muslim physicians is required to enrich the patient-centered approach. This study was limitedby the absence of earlier literature discussing pre-Ramadan consultation, so further work is needed to cover the literature gap.

## Introduction

A leading belief in Islam is to do no self-harm [1]. Therefore, a child who has not achieved puberty, pregnant women, and patients with some chronic diseases that may deteriorate with fasting are excused from fasting [2]. Despite this, studies show that numerous Muslim patients with chronic diseases opt to fast during Ramadan despite uncontrolled medical conditions [3]. According to a survey that included 12,000 Muslim diabetic patients, 79% (9480) decided to fast despite having uncontrolled diabetes [4]. Although fasting has several health benefits, it may pose risks to patients with existing medical issues [5]. Therefore, Ramadan fasting represents a challenge to Muslim patients and their healthcare professionals [3].

Research has shown that patients with chronic medical conditions require counseling before Ramadan [6]. Ideally, counseling is held one to four months before the start of fasting [7]. The pre-Ramadan consultation includes determining individual risks for fasting, patients' stratification, and shared decision-making [8]. As such, the advice would be: (i) strong advice against fasting, (ii) advice against fasting, or (iii) permission to fast [6]. This approach plays a role in perfecting the care of patients with chronic conditions during the fasting month.

Nevertheless, no study has been designed to assess the uptake of pre-Ramadan consultation guidelines. We could not find any published literature on pre-Ramadan counseling or its use among healthcare professionals. Thus, the approach of healthcare professionals to pre-Ramadan consultation is not clear. The existing literature discusses the management guidelines for multiple medical conditions during Ramadan [9,10]. In fact, the findings of some studies underpin a knowledge gap among physicians, in which doctors did not feel comfortable counseling and managing the health of these patients [5,11]. However, none of them focused on pre-Ramadan counseling strategies.

The present study aims to assess healthcare professionals' knowledge, attitude, and practice (KAP) toward pre-Ramadan counseling in Arab countries. Another aim was to determine whether physicians' specialties and religious beliefs influence their utilization of pre-Ramadan consultations. For this purpose, we surveyed primary care physicians to estimate the use rate and understand its influencing factors. We hypothesized that KAP toward pre-Ramadan consultation differs among physicians according to their religious beliefs and specialties.

## Materials and methods

Study design

This cross-sectional survey was conducted between February and March 2022 to assess the KAP toward pre-Ramadan consultation and management of patients with pre-existing health issues before Ramadan.

Material

The primary outcome measures were knowledge, attitude, and physicians' practice toward pre-Ramadan consultation. The secondary outcome measure was the influence of participants' specialty and religious beliefs on KAP and the use of pre-Ramadan consultation.

The practice guideline about pre-Ramadan consultation published in the British Medical Journal (BMJ) in 2022 [8] was used to measure physicians' KAP. A 32-item questionnaire was developed, and its internal consistency was adequate, with a Cronbach's alpha of 0.83 throughout all elements [12]. The questions were under four headings: (i) demographics; (ii) knowledge; (iii) attitude; and (iv) practice. The questionnaire was assessed and reviewed by five family medicine consultants. The researchers adjusted the wording of the questionnaire based on the physicians’ feedback

Measuring knowledge

Knowledge was assessed based on the BMJ practice guidelines [8]. So, participants were asked about their medical advice regarding fasting during Ramadan for a patient with pre-existing medical conditions (e.g., patient with advanced heart failure (left ventricular ejection fraction (LVEF) <35%, New York Heart Association (NYHA) III-IV)). The participants indicated their advice by selecting one of the following: strong advice against fasting; advice against fasting; patient may fast; referral to specialists; and no answer [6]. The maximum score for the knowledge section that could be attained by participants was 17.

Measuring attitude

In the attitude section, participants were asked to indicate their level of agreement with each statement on a three-point scale (disagree, neutral, agree). For example, healthcare professionals should actively identify and advise patients with pre-existing conditions about fasting before Ramadan. The maximum score for the attitude section that could be attained by participants was four.

Measuring practice

Participants were asked about their practices regarding pre-Ramadan consultation and in how many patient visits the physician adheres to pre-Ramadan guidance [6]. The participants indicated their frequency of practicing counseling by selecting one of the following: 1-24% of patients' visits, 25-49% of patients' visits, 50-74% of patients' visits, >75% of patients' visits (e.g., "Determine the individual risk for fasting before Ramadan."). The maximum score for the practice section that could be attained by participants was 11.

Population and recruitment

Participants' specialties covered the fields of family medicine (received a structured residency program), general practitioner, internal medicine, and obstetrics and gynecology. Throughout this paper, the term "physicians" will refer to family medicine and general practitioner, internal medicine, and obstetrics and gynecology, and the term "Arab countries" will refer to Bahrain, Saudi Arabia, Lebanon, and Qatar.

The participants were from multiple healthcare centers across the Kingdom of Bahrain, Saudi Arabia, Lebanon, and Qatar. The researchers identified 350 doctors from different medical centers. The list was from three hospitals namely, American Mission Hospital, Bahrain; Primary Healthcare Corporate Center, Qatar; and Prince Sultan Military Hospital. Potential participants were invited to fill it out online using LimeSurvey survey tool (LimeSurvey GmbH, Hamburg, Germany). The potential participant received two personalized reminders over WhatsApp (Whatsapp LLC, Menlo Park, California, United States. Informed consent was taken before completing the online survey. Research and Ethics Committee in the American Mission Hospital, Manama, Bahrain gave approval for the study (approval number: 03/2022)

For our study, a sample size of n=208 would ensure that a two-sided test with a=0.05 had 90% power to a 30% difference in the proportion of physicians giving pre-Ramadan counseling. The final sample satisfied these requirements. G*Power software was used [13]. Two hundred doctors filled out the survey.

Statistical analyses

Each participant got three scores: (1) a knowledge score, (2) an attitude score, and (3) a practice score. The "knowledge score" was calculated according to the number of correct responses among the 17 knowledge queries about pre-Ramadan consultations and the management of chronic medical conditions before Ramadan. Likewise, each participant received an attitude score according to the number of positive responses among the four attitude questions about pre-Ramadan consultation. Similarly, each participant's practice score was calculated according to the number of correct practices among the 11 queries about pre-Ramadan consultation.

Data was collected through LimeSurvey and was exported to IBM SPSS Statistics for Windows, Version 26.0 (Released 2019; IBM Corp., Armonk, New York, United States) for analysis. Incomplete surveys were excluded. Descriptive analyses were applied for demographic items. A one-way ANOVA and post hoc tests were performed to detect the differences in physicians' KAP toward pre-Ramadan consultation with their specialties and religious backgrounds. The test was two-sided, and a p-value less than 0.05 was considered statistically significant.

## Results

A total of 350 physicians were invited to complete the survey, and 253 questionnaires were received. Of these, 53 surveys were excluded because of incomplete data. Data analysis was conducted on the remaining 200 questionnaires (effective response rate of 79%). Of the 200 responses, 53.5% of the participants were female, and 46.5% were male. The average age was 42 years, with a standard deviation of 8.3.

Most of the participants (84%) had not received training for pre-Ramadan consultation. At the same time, most participants reported initiating the consultation one month before Ramadan (93%), and only two participants (out of 200) initiated pre-Ramadan consultations at least three months before the fasting month. The mean value of the knowledge score was 7.87 (SD 2.926) out of 17. Next, the average positive attitude score was 2.28 (SD 1.147) out of 4. Finally, physicians' average correct practices score was 4.33 (SD 2.60) out of 11. Table 1 presents the breakdown of participants according to country of practice, specialty, type of practice, and religion.

**Table 1:** Demographic characteristics of participants (N=200) and scores M: Mean; SD: Standard Deviation

Sample Characteristics	N (%)	M (SD)
Gender		
Male	93 (46.5%)	
Female	107 (53.5%)	
Age		42 (8.3)
Country		
Bahrain	84 (42%)	
Qatar	33 (16.5%)	
Saudi Arabia	49 (24.5%)	
Lebanon	34 (17%)	
Specialty		
Family Medicine	82 (41%)	
General Practice	47 (23.5%)	
Internal Medicine	36 (18%)	
Obstetrics & Gynecology	35 (17.5%)	
Type of Practice		
Major City	126 (63%)	
Sub-Urban	49 (24.5%)	
Rural	25 (12.5%)	
Religion		
Muslim	111 (55.5%)	
Christian	36 (18%)	
Hindu	31 (15.5%)	
Buddhism	22 (11%)	
Have you received training about Per-Ramadan?		
Yes	32 (16%)	
No	168 (84%)	
When participants provide Pre-Ramadan consultation		
Before one month of Ramadan	186 (93%)	
Before two months of Ramadan	12 (6%)	
Before three months of Ramadan	2 (1%)	
Scores of physicians		
Pre-Ramadan consultation Knowledge score	200	7.87 (2.926)
Pre-Ramadan consultation attitude score	200	2.28 (1.147)
Pre-Ramadan consultation practice score	200	4.33 (2.60)

Table 2 shows the mean difference in physicians’ scores with different specialties. One-way ANOVA showed a statistically significant difference in the mean value of knowledge scores with different specialties (f(3,196) =6.303, p < 0.001). There was no statistically significant difference in the mean attitude and practice scores between specialties (f(3,196) =1.444, p= 0.231, f(3,196) =0.812, p =0.489, respectively. A Games-Howell post hoc test for multiple comparisons found that the mean value of knowledge scores was significantly different between family physicians and: general practitioners (p = 0.002, 95%CI = 0.51, 3.12), internists (p = 0.01, 95%CI = 0.32,3.15), and obstetricians (p = 0.03, 95%CI = 0.12, 3.28) (Table 3)

**Table 2:** ANOVA of knowledge, attitude, and practice by specialty

		Sum of Squares	df	Mean Square	F	p-value
Knowledge Score	Between Groups	149.876	3	49.959	6.303	0.0001
	Within Groups	1553.479	196	7.926		
	Total	1703.355	199			
Attitude Score	Between Groups	5.664	3	1.888	1.444	.231
	Within Groups	256.211	196	1.307		
	Total	261.875	199			
Practice Score	Between Groups	10.358	3	3.453	.812	.489
	Within Groups	833.862	196	4.254		
	Total	844.220	199			

**Table 3:** Multiple comparison dependent variable: knowledge score *. The mean difference is significant at the 0.05 level.

(I) Specialty	(J) Specialty	Mean Difference (I-J)	Standard Error	p-value		
					Family Medicine	General Practice	1.817^*^	.501	.002		
Internal Medicine	1.736^*^	.537	.010			
OB & GYN	1.702^*^	.599	.030			
General Practice	Family Medicine	-1.817^*^	.501	.002		
	Internal Medicine	-.082	.568	.999		
	OB & GYN	-.115	.626	.998		
Internal Medicine	Family Medicine	-1.736^*^	.537	.010		
	General Practice	.082	.568	.999		
	OB & GYN	-.033	.656	1.000		
OB & GYN	Family Medicine	-1.702^*^	.599	.030		
	General Practice	.115	.626	.998		
	Internal Medicine	.033	.656	1.000		

Table 4 shows the mean difference in physicians’ scores with respect to different religions. One-way ANOVA showed a statistically significant difference in the mean value of attitude and practice scores with different religions (f (3,196) =10.782, p < 0.001), (f (3,196) =11.743, p < 0.001), respectively. There was no statistically significant difference in the mean knowledge score among religious groups (f (3,196) =1.301, p= 0.275). A Games-Howell post hoc test for multiple comparisons found that the mean value of attitude scores and practice scores were significantly different among physicians with different religious backgrounds. Statistically, the p-value for attitude scores was significant for Muslim physicians as compared to Christian (p = 0.026, 95%C.I. = 0.6, 1.31), Hindu (p = 0.004, 95% C.I. = 0.23, 1.48), and Buddhist physicians (p = 0.02, 95%CI = 0.12, 1.69). Similarly, multiple comparisons found that the mean value of practice scores was significantly different for Muslim physicians as compared to Christian (p = 0.029, 95%CI = -2.12, -0.8), Hindu (p = 0.003, 95%CI = -0.2.88, -0.47), and Buddhist physicians (p = 0.002, 95%CI = -3.42, -0.65) (Table 5)

**Table 4:** ANOVA of knowledge, attitude, and practice toward pre-Ramadan consultation by religious belief

		Sum of Squares	df	Mean Square	F	p-value
Knowledge Score	Between Groups	33.250	3	11.083	1.301	.275
	Within Groups	1670.105	196	8.521		
	Total	1703.355	199			
Attitude Score	Between Groups	32.347	3	10.782	9.207	.0001
	Within Groups	229.528	196	1.171		
	Total	261.875	199			
Practice Score	Between Groups	128.626	3	42.875	11.743	.0001
	Within Groups	715.594	196	3.651		
	Total	844.220	199			

**Table 5:** Multiple comparison dependent variable: attitude and practice toward pre-Ramadan consultation by religious belief *. The mean difference is significant at the 0.05 level.

Dependent Variable	(I) Religion	(J) Religion	Mean Difference (I-J)	Std. Error	P value	
						Attitude Score	Muslim	Christian	.686^*^	.235	.026	
Hindu	.856^*^	.233	.004				
Buddhism	.903^*^	.286	.020				
Christian	Muslim	-.686^*^	.235	.026		
	Hindu	.170	.307	.945		
	Buddhism	.217	.349	.925		
Hindu	Muslim	-.856^*^	.233	.004		
	Christian	-.170	.307	.945		
	Buddhism	.047	.348	.999		
Buddhism	Muslim	-.903^*^	.286	.020		
	Christian	-.217	.349	.925		
	Hindu	-.047	.348	.999		
Practice Score	Muslim	Christian	-1.101^*^	.383	.029	
		Hindu	-1.674^*^	.448	.003	
		Buddhism	-2.033^*^	.505	.002	
	Christian	Muslim	1.101^*^	.383	.029	
		Hindu	-.573	.548	.724	
		Buddhism	-.932	.595	.408	
	Hindu	Muslim	1.674^*^	.448	.003	
		Christian	.573	.548	.724	
		Buddhism	-.359	.639	.943	
	Buddhism	Muslim	2.033^*^	.505	.002	
		Christian	.932	.595	.408	
		Hindu	.359	.639	.943	

## Discussion

The goal of the present study was to assess physicians' KAP toward pre-Ramadan consultation and investigate whether physicians' specialties and religious backgrounds influence their KAP.

Our findings converge with previous findings that physicians have a low level of knowledge about managing chronic diseases during Ramadan [5,7]. In addition, they show that only 1% of physicians in Arab countries use pre-Ramadan consultation on time, which is three to four months before Ramadan. A possible explanation for this might be the lack of training among most physicians (84%) in this study. Family physicians were the most knowledgeable in pre-Ramadan consultation compared with other specialties. Our results agree with other studies, in which family physicians were more likely to ask about patients' religion and the role of belief in the management of illnesses [14].

It is interesting to note that religious backgrounds did not influence physicians' knowledge scores. However, non-Muslim doctors got lower scores in attitude and practice than Muslim doctors. These results seem to be consistent with other research, which found that physicians' religious beliefs could influence their practice [15], and that modern doctors avoid discussing topics related to religious beliefs with their patients [16]. Moreover, these results are likely to be explained by Muslim participants benefiting from their inherited awareness of Ramadan rules and their experience with fasting scenarios.

Our findings offer a novel perspective: pre-Ramadan consultation is underutilized across medical specialties, which carries a substantial risk for patients with chronic diseases who wish to fast during Ramadan. Moreover, it is a unique study highlighting the influence of physicians' specialties and religions on their KAP toward pre-Ramadan consultation. This study underpins the importance of patient-centered care by improving the attitude and practice of non-Muslim doctors toward Pre-Ramadan consultation.

Therefore, the present study highlights the importance of training physicians about pre-Ramadan consultation and providing exceptional attention to non-Muslim physicians (e.g., a lecture about Islamic opinions toward fasting in Ramadan) to improve the patient-centered approach.

The most critical limitation is that the study has suffered from a lack of well-grounded theoretical considerations. To date, pre-Ramadan consultation has received scant attention in the research literature. However, this study highlights literature gaps, and further studies need to be conducted to validate the instrument used in this study and explore patient attitudes toward pre-Ramadan consultation.

## Conclusions

Although this study has shown that family physicians were the most knowledgeable compared with other specialties, the data has found a low degree of knowledge regarding pre-Ramadan consultation. Most participants did not use pre-Ramadan consultation timely. The second significant finding was that the attitude and practice toward pre-Ramadan consultation were statistically different between Muslim and non-Muslim doctors. The findings of this study suggest that improving physicians' pre-Ramadan consultation knowledge is imperative to optimizing the care of patients before Ramadan. Moreover, improving the attitude and practice of non-Muslim physicians is required to improve the patient-centred approach. This study was limited by the absence of previous literature discussing pre-Ramadan consultation, so further work needs to be done to establish whether pre-Ramadan consultations affect patients' fasting decisions.
